# Exploring the links between water, sanitation and hygiene and disability; Results from a case-control study in Guatemala

**DOI:** 10.1371/journal.pone.0197360

**Published:** 2018-06-01

**Authors:** Hannah Kuper, Islay Mactaggart, Sian White, Carlos Dionicio, Rafael Cañas, Jonathan Naber, Sarah Polack, Adam Biran

**Affiliations:** 1 International Centre for Evidence in Disability, London School of Hygiene & Tropical Medicine, London, United Kingdom; 2 Department of Disease Control, London School of Hygiene & Tropical Medicine, London, United Kingdom; 3 National Council on Disability, Guatemala City, Guatemala; University of Pittsburgh, UNITED STATES

## Abstract

**Objective:**

To assess the Water, Sanitation and Hygiene (WASH) access and appropriateness of people with disabilities compared to those without, in Guatemala.

**Methods:**

A case-control study was conducted, nested within a national survey. The study included 707 people with disabilities, and 465 age- and sex-matched controls without disabilities. Participants reported on WASH access at the household and individual level. A sub-set of 121 cases and 104 controls completed a newly designed, in-depth WASH questionnaire.

**Results:**

Households including people with disabilities were more likely to use an improved sanitation facility compared to control households (age-sex-adjusted OR: 1.7, 95% CI 1.3–2.5), but otherwise there were no differences in WASH access at the household level. At the individual level, people with disabilities reported greater difficulties in relation to sanitation (mean score 26.2, SD 26.5) and hygiene access and quality (mean 30.7, SD 24.2) compared to those without disabilities (15.5, 21.7, p<0.001; 22.4, 19.1, p<0.01). There were no differences in different aspects of water collection between people with and without disabilities in this context where over 85% of participants had water piped into their dwelling. Among people with disabilities, older adults were more likely to experience difficulties in hygiene and sanitation than younger people with disabilities.

**Conclusions:**

People with disabilities in Guatemala experience greater difficulties in accessing sanitation facilities and practicing hygienic behaviours than their peers without disabilities. More data collection is needed using detailed tools to detect these differences, highlight which interventions are needed, and to allow assessment of their effectiveness.

## Introduction

Access to Water, Sanitation and Hygiene (WASH) is an essential component of living a healthy life, and lack of access to WASH can have broad ranging health and social consequences.[1, 2] Exclusion from WASH facilities still remains common, as 2.4 billion people lack access to adequate sanitation, and 663 million use unimproved water supplies or surface water.[3] Consequently, a key Sustainable Development Goal (SDG) is to “Ensure availability and sustainable management of water and sanitation *for all*” (emphasis added). However, people with disabilities face wide-ranging discrimination and exclusion that may lead to additional difficulties with access to WASH. [4] This is an important issue to explore, since there are an estimated one billion people in the world with disabilities, equating to one in seven people worldwide.[4] Their inclusion in WASH provision is therefore essential if we are to achieve the WASH-related SDG.

The qualitative literature shows that WASH is a major life concern among people with disabilities,[5] [6] and can contribute to exclusion in education and in the workplace. [7] [8] However, quantitative data are lacking on the relationship between WASH access and disability,[5] as well as evidence on how to improve inclusion. [9] There are two main pathways for the association between WASH access and disability. At the household level, people with disabilities may be more likely to live in households that are poorer,[10] and therefore lack adequate WASH access. At the individual level, people with disabilities may face a range of barriers that make it more difficult to access WASH facilities that are available to other household members. For instance; people with disabilities may find it physically difficult to access small latrines or water points with steps (physical barriers), they may not be invited to participate in WASH-related community events or the information provided may be inaccessible (institutional barriers) and/or they may face stigma leading to exclusion from facilities (social barriers).[6]

There is another issue beyond access to WASH facilities that has received little attention, and this is the question of whether the WASH facilities available are appropriate for the specific needs of people with disabilities. A qualitative study in Malawi highlighted that even when people with disabilities are able to access WASH facilities, they may experience difficulties in using these facilities in the same way as other household members, for example without assistance, without pain and without loss of dignity. [6] Issues about appropriateness and quality of WASH for people with disabilities are rarely assessed by existing quantitative tools that measure WASH access.

In order to address these gaps in knowledge, we developed a survey tool for collecting quantitative data on the access and quality of WASH in relation to disability. Our intention was to create a quantitative tool that would allow comparable data to be collected in different settings to address the following questions: Is the level of access to water and sanitation worse for people with disabilities than people without disabilities, and is the quality of access to water and sanitation poorer for people with disabilities than for those without?. We pilot-tested this tool within a national case-control study of disability in Guatemala in order to assess its feasibility. Guatemala is a middle-income country, with a population of 16.6 million people. Overall, there is relatively good WASH coverage in Guatemala; A 2017 report shows that 94% of Guatemalans have access to at least basic water supplies (89% in rural areas), 67% to at least basic sanitation (53% in rural areas) and 77% to at least basic water and soap for hygiene (70% in rural areas). [3] This setting therefore allows us to explore issues of access and quality of WASH for people with disabilities, as coverage is relatively good. Disability is common in Guatemala; A recent national survey in Guatemala estimated the prevalence of disability at 10.2%, and that people with disabilities were on average older, poorer, less likely to be employed and had worse access to education. [11] The information generated from this study could be used to plan and advocate for more appropriate WASH services for people with disabilities in Guatemala, as well as inform data collection on WASH for people with disabilities more generally.

## Materials and methods

### Study design

A population-based case-control study was conducted, nested within the national survey of disability in Guatemala.[11]

### National survey

The case-control study was conducted within the context of a national survey of disability across all 22 departments of Guatemala. Multi-stage, stratified cluster-random sampling with probability proportional to size procedures was used to identify a nationally representative sample, using the 2012 Census as the sampling frame. We randomly selected 56 clusters (enumeration areas) within each of the five regions in Guatemala. Within each cluster, we used compact segment sampling to divide the cluster into equal segments of approximately 50 people (10 households). One segment was randomly selected and all households were visited door to door, until 50 people had been included.

Within each household the purpose of the survey was explained verbally to the household head or an adult key informant and we obtained permission from all household members included in the survey. Demographic data (age, sex, ethnicity and adults: education, literacy and marital status) were collected on all household members. In addition, household-level data on indicators of socio-economic status (SES) were recorded through questions (ownership of assets) and observation (building materials the house).

Disability status was assessed for each household member aged ≥2 years using the Washington Group (WG) Extended Set on Functioning for adults (≥18 years), and the UNICEF/WG Extended Set on Functioning for children (2–17 years). [12] Children aged <2 years were excluded due to the lack of available survey tools to assess disability in this age group.

Disability was defined as reporting “significant” functional limitations in at least one domain, namely:

- Adults:
  ○ reporting “a lot of difficulty” or “cannot do” in seeing, hearing, walking, self-care, communication (understanding/being understood), cognition (remembering and concentrating), upper body (fine motor dexterity and upper body strength)
  ○ For anxiety and depression domains: reporting experience of anxiety/depression daily and at the level of ‘a lot’
- Children:
  ○ Aged 2–4: Reporting “a lot of difficulty” or “cannot do” in seeing, hearing, walking, fine motor dexterity, understanding, being understood. Learning, playing and controlling behaviour
  ○ Aged 5–17: Reporting “a lot of difficulty” or “cannot do” in seeing, hearing, walking, self-care, understanding, being understood, learning, remembering, concentrating, accepting change, controlling behaviour, anxiety and depression

### Recruitment of cases and controls

All people with a disability (as defined above) identified during the course of the national survey were included in the nested case-control study. For each person identified as having a disability (“case”) one age and sex matched “control” who did not fulfil the case criteria was selected from within the same cluster. Controls were matched by age within +/-10 years for adults (aged 18+ years) and +/- 2 years for children (aged 2–17 years).

### Data collection

Informed written consent was sought from all participants in the nested case-control. Participants <18 or for whom it was impossible or inappropriate to obtain consent directly (e.g. people with severe cognitive impairments) were asked for verbal assent, with written consent given by the caregiver or guardian, who remained throughout the interview process. Any individuals who expressed discomfort about participation were excluded from the study.

Interviews were conducted in Spanish or in the dominant Mayan languages (Mam, K’iche’, Kaqchikel, and Q’eqchi). For children under 10 years, questions were asked of the child’s primary caregiver, in the presence of the child where possible. Children aged 10–17 years were interviewed directly in the presence of an adult caregiver. For any child aged 10–17 years or adult (≥18years) who was unable to communicate independently, questions were asked of an adult caregiver as a proxy.

All cases and controls were interviewed using standardised questionnaires. These included questions about Water (4 questions–covering source of drinking water, distance to drinking water and ability to access drinking water when needed) and Sanitation (8 questions–covering type of toilet facilities available at household level, and used by individual, and whether this facility could be used without assistance and without contact with faeces). Household water and sanitation facilities were classified as improved as follows: [3]

- Improved sanitation: flush/pour flush to piped sewer system, septic tank or latrine pit; ventilated improved pit latrines, composting toilets or pit latrines with slabs.
- Improved water source: piped water, boreholes or tubewells, protected dug wells, protected springs, and packaged or delivered water.

A further module on appropriateness of WASH was developed by three authors (HK, AB, SW) based on findings of their previous qualitative research in Malawi,[6] and learning from previous tools that have been developed to assess different aspects of WASH.[13, 14] This module included further details on Water (7 questions), Sanitation (5 questions) and Hygiene (10 questions) specifically relevant to the needs of people with disabilities, with binary responses of “yes” or “no” (Described in S1 Table). An additional question on menstrual hygiene management was added for women aged 15 to 49. The module was reviewed by four sector experts for comments (academics, practitioners), and revised prior to use. The module was translated into Latin American Spanish and reviewed by the Guatemala research team to ascertain that the questions were appropriate and understandable. Subsequently, the module was introduced in the current study within two regions of Guatemala (North East and South East Guatemala), and so was available for a sub-set of the total population screened.

Further questionnaire sets were included to measure health, livelihood, education and participation, but these measures were not used in the current analyses.

### Team and training

Five survey teams, two comprising three interviewers and three comprising four interviewers conducted the fieldwork. Interviewers underwent a ten-day training on the project protocol and methods.

### Ethics

Ethical approval for the study was provided by: the London School of Hygiene & Tropical Medicine (LSHTM) and the Comité de Ética Independiente en Investigación (Latin Ethics), Guatemala. A National Directory of Disability Services was compiled with support from Asociación de Asistencia Técnica y Capacitación en Educación y Discapacidad (ASCATED), CBM and the National Council for the Care of Persons with Disabilities (CONADI), and distributed to the nearest public health service to each of the study clusters. We advised participants expressing desire for services in relation to disability to visit their nearest health service.

### Data analysis

All data were collected on android tablets using a bespoke mobile application. Data from the tablets were transferred daily via Wi-Fi to a secure, password-protected, cloud-based server.

Data analysis was completed using the statistical package STATA. We constructed an SES score using principal component analysis (PCA). PCA involves a statistical calculation of the relative weight of different assets (household characteristics and working durable assets such as vehicles or white goods) producing a total score per household The principal component on which the socio-economic status (SES) index was derived comprised an eigenvalue of 6.12 and explained 21% of the variance, supporting its suitability in representing SES (data not shown). This SES score was then divided into quartiles.

Multivariable logistic regression analysis was used to identify differences in WASH access between people with and without disabilities, adjusted for age, sex, region and SES.

Three additive summary scores for sanitation, water and hygiene were constructed based on available following items, which had Yes/no response options (S1 Table). Each score was transformed into a score out of 100, with 100 equating to the maximum total difficulties and 0 to no difficulties, so that higher scores reflected a greater degree of difficulties. Multivariate linear regression was then used to compare the scores between cases and controls, adjusted for age, sex, SES and region. Scores were only applied to participants where the further detailed WASH module was completed (i.e in the North East and South East Regions), and the water score was only applied to those who did not have water piped into their homes.

## Results

In total, 14,873 people were enumerated for the survey and 13,073 were screened for disability (88%). The study participants were representative of the national population in terms of age and sex distribution (S2 Table). The survey identified 707 people with disabilities and 465 age- and sex matched controls without disabilities. People with disabilities were significantly older than those without, but were well-matched on sex (64% female among cases and 65% among controls) (Table 1). There were some differences between people with and without disabilities in regional distribution but no significant differences in SES. The most common functional limitations among the people with disabilities were anxiety/depression (44%) followed by physical (31%) and seeing (28%) difficulties.

**Table 1:** Socio-demographic characteristics of people with and without disabilities*.

	People with disabilities (n = 707)		People without disabilities (n = 465)		Age, Sex, adjusted OR (95% CI)
	N	(%)	N	(%)	
**Age**					
5–14	95	13	79	17	0.4 (0.2–0.6)
15–24	96	14	103	22	0.3 (0.2–0.4)
25–54	266	38	182	39	0.5 (0.3–0.7)
55–64	80	11	47	10	0.5 (0.3–0.9)
65+	170	24	54	12	Baseline
**Sex**					
Male	253	36	163	35	Baseline
Female	454	64	301	65	1.1 (0.8–1.4)
**Region**					
Central	194	27	123	26	Baseline
North-East	66	9	50	11	0.7 (0.4–1.0)
North-West	233	33	110	24	1.3 (1.0–1.8)
South-East	55	8	54	12	0.5 (0.3–0.8)
South-West	159	22	128	28	0.7 (0.5–1.0)
**SES**					
1^st^ Quartile (poorest)	155	22	116	25	0.9 (0.6–1.2)
2^nd^ Quartile	198	28	120	26	1.1 (0.8–1.5)
3^rd^ Quartile	182	26	118	25	1.0 (0.7–1.4)
4^th^ Quartile (richest)	172	24	111	24	Baseline
**Functional limitation type**					
Seeing	200	28	-		-
Hearing	104	15	-		-
Physical	218	31	-		-
Anxiety/Depression	314	44	-		-
Self-care	80	11	-		-
Cognition/Communication	69	10	-		-

*This table is adapted from tables in the ENDIS report. [11]

Table 2 summarises access to WASH among households including at least one member with a disability compared to households without people with disabilities from the national sample. Most households had an improved sanitation facility, but this was significantly more common in households including a person with disability (89%) compared to control households (84%, age-sex- adjusted OR: 1.7, 95% CI 1.3–2.5). Otherwise, there were no differences in access to sanitation or water facilities at the household level.

**Table 2:** Access to WASH at household level among people with disabilities compared to people without disabilities.

Household level variables	Households including people with disabilities (n = 707)		Households not including people with disabilities (n = 465)		Age, Sex, Region, SES adjusted OR (95% CI)
	N	(%)	N	(%)	
**Type of Sanitation Facility**					
Improved	630	89%	391	84%	1.7 (1.3–2.5)
Unimproved/ no facility	77	11%	74	16%	Baseline
**Location of Sanitation Facility** Household	651	92%	427	92%	Baseline
Shared	53	8%	35	8%	1.2 (0.7–1.8)
Public	3	1%	3	1%	0.9 (0.2–4.8)
**Water Facility**					
Improved	695	98%	450	97%	Baseline
Unimproved	12	2%	15	3%	0.6 (0.3–1.4)
**Distance to Water Source**					
Within the house	612	87%	394	85%	Baseline
0–10 minutes	74	10%	52	11%	1.0 (0.7–1.5)
>10 minutes	21	3%	19	4%	0.7 (0.4–1.4)

Within households, people with disabilities reported greater difficulties using the same sanitation facility as other household members (Table 3). On prompting for reasons for greater difficulty, 59% reporting that it would be physically impossible for them to do so. Furthermore, people with disabilities were less likely to be able to use the facilities without assistance from others compared to those without disabilities (OR = 0.6, 95% CI 0.4–0.8). Nearly nine times as many with disabilities reported that they had to make adjustments in their daily routine/practices in the use of toilets, as compared to those without (8.9, 95% CI = 2.7–30.0) although numbers were small for this comparison. These changes included limiting fluid intake (16% of people with disabilities), sometimes soiling self (16%), using special clothing (16%) and/or limiting food intake (14%). Most respondents had water piped into their household (87% of cases, 85% of controls) and consequently there were no differences detected in water-collection or water-access between people with and without disabilities.

**Table 3:** Access to WASH among people with disabilities compared to people without disabilities*.

	People with disabilities (n = 707)		People without disabilities (n = 465)		Age, Sex, Region, SES adjusted OR (95% CI)
	N	(%)	N	(%)	
**Individual level variables**					
**Use same facility as other household members** Yes	666	94%	460	99%	0.2 (0.1–0.4)^§^
**Use facility without faecal contact**					
Yes	500	71%	352	76%	0.8 (0.6–1.0)
**Use facility without assistance from others**					
Yes	528	75%	389	84%	0.6 (0.4–0.8) ^§^
Make changes in daily routine/practices in use of toilet^**^					
Yes	36	5%	3	1%	8.9 (2.7–30.0) ^§^
Collect water for drinking***					
Yes	194	73%	154	83%	0.7 (0.4–1.2)
**Able to access drinking water when need it**					
Yes	616	87%	405	87%	1.0 (0.7–1.4)

*This table is adapted from tables in the ENDIS report. [11]

**Data for regions 2 and 4 only

***Excludes all participants for whom water is piped into the dwelling

§ p<0.05

A sub-set of 121 cases and 104 controls from two regions of Guatemala (North East and South East Guatemala) completed the in-depth questionnaire about WASH access appropriateness and quality (Table 4). The tool appeared to work relatively well, without missing data, a high spread of positive and negative answers, and good discrimination between people with and without disabilities in hygiene and sanitation access. People with disabilities reported more pain when using sanitation facilities compared to people without disabilities (3.6, 95% CI 1.5–8.6), but no differences on other individual sanitation questions. Overall, the sanitation score suggests difficulties in this domain were greater for people with disabilities (mean = 26.2, SD = 26.5) than those without (15.5, 21.7) (p-value <0.01). There were no significant differences in different aspects of water collection between those with and without disabilities, although the sample size for this comparison was small as it was restricted to people whose water source was not piped into the dwelling (27 cases and 28 controls). In terms of hygiene, people with disabilities reported needing more help when bathing (4.8, 95% CI 2.3–10.1) and more pain when bathing (6.9, 95% CI 1.5–32.7) than those without disabilities, although the numbers of controls reporting pain when bathing was small. People with disabilities also reported that they were less likely to be able to use the same place for bathing as other members of the household (0.3, 95% CI 0.1–0.7) and experienced greater difficulty in finding soap or cleansing materials without help from others (0.4, 95% CI 0.2–0.8). People with disabilities were more likely to come into contact with dirt or dirty water when bathing compared to controls (2.0, 95% CI 1.1–3.8). Overall, the hygiene score suggests difficulties in this domain were significantly more common for people with disabilities (mean = 30.7, SD = 24.2) compared to those without (22.4, 19.1, p value<0.05). There were no differences around management of menstruation, although the numbers were small for this comparison (6 women with disabilities and 7 women without disabilities).

**Table 4:** Inclusive WASH assessment among people with disabilities compared to people without disabilities from two regions of Guatemala (North East and South East Guatemala).

	People with disabilities (n = 121) N (%)		People without disabilities (n = 104) N (%)		Age, Sex, Region, SES adjusted OR (95% CI)
Sanitation					
**Causes additional pain to use the sanitation facility usually used**Yes	30	28	8	10	3.6 (1.5–8.6)***
**Have as much privacy as other members of your household when you go for defecation**	85	79	64	76	1.4 (0.6–2.8)
**Fear physical or verbal violence when going for defecation**	13	12	9	11	1.1 (0.4–3.0)
**Able to use the toilet facility without you or your clothes coming into contact with urine**	62	58	53	63	0.8 (0.5–1.6)
**Sanitation Score (mean, SD)**	26.2 (26.5)		15.5 (21.7)		<0.01**
Water*					
**Need help from others**	9	33	9	32	1.2 (0.3–4.5)
**Can use same source as other household members**	26	93	28	100	-
**Can collect same quantity as other household members**	20	74	23	82	0.5 (0.01–2.6)
**Cause additional pain to collect water**	11	41	9	32	2.0 (0.4–9.3)
**Fear physical or verbal violence collecting water**	2	7	2	7	2.7 (0.2–37.9)
**Water Score (mean, sd)**	30.7 (19.7)		23.0 (14.7)		0.4**
**Hygiene**					
**Bathing place not in house, compound or yard**	13	12	13	15	0.8 (0.4–2.0)
**Usually need help from others when going for bathing**	49	46	13	15	4.8 (2.3–10.1)***
**Use the same place for bathing as other members of the household**	85	79	79	84	0.3 (0.1–0.7)***
**Causes additional pain to use this place to bathe**	16	15	2	2	6.9 (1.5–32.7)***
**Have as much privacy as other members of your household when you bathe**	91	85	71	85	1.0 (0.4–2.4)
**Fear physical or verbal abuse when bathing**	8	7	5	6	1.6 (0.4–5.5)
**Use this place without coming into contact with dirt or dirty water**	61	57	33	39	2.0 (1.1–3.8)***
**Can wash your hands without help from others**	88	82	70	83	1.0 (0.4–2.3)
**Can locate and use soap or other cleansing materials without help from others**	77	72	755	89	0.4 (0.2–0.8)***
**Hygiene Score (mean, sd)**	30.7 (24.2)		22.4 (19.1)		<0.05
**Often get blood on clothing when menstruating**	6	14	7	15	0.9 (0.3–3.3)

*Restricted to those who collect water for drinking and who’s water source is not piped into the dwelling (27 cases and 28 controls)

**p-value from multivariate linear regression

***p-value<0.05

Among people with disabilities, sanitation, water and hygiene scores (ranging from 0- no difficulty to 100 –highest level of difficulty) were compared between men and women, different age-groups, rural-urban location and by type of functional limitation(Table 5). Reported difficulties in hygiene and sanitation were lowest among children (12.8, 8.0 respectively) and increased in adults (20.4, 28.9) and older adults (28.2, 35.5, p-value <0.01 for both comparisons). Water scores did not vary by age, and sanitation, water or hygiene scores differ between men and women. Water scores were worse in rural than urban areas, but there were no differences in hygiene or sanitation scores by location. There was little variation in difficulties in sanitation or hygiene by functional domain, although the numbers in sub-groups were relatively small. For water, reported difficulties were greatest for people with difficulties in seeing (45.9) and cognition (40.5), and lowest for those with difficulties in walking (17.9), with overlapping confidence intervals between these disability type sub-groups.

**Table 5:** Inclusive WASH assessment among people with disabilities, by age, sex and domain of functional limitation from two regions of Guatemala (North East and South East Guatemala).

	N	Sanitation scores (95% CI)	Water scores (95% CI)	Hygiene scores (95% CI)
Age				
• Child (<18 years)	28	12.8 (4.7–19.8)	-	8.0 (1.5–14.5)
• Adult (18–59)	68	20.4 (14.5–26.2)	33.3 (20.6–46.0)	28.9 (24.1–33.6)
• Older Person (60+)	75	28.2 (22.0–34.3)	30.8 (19.8–41.7)	35.5 (30.1–40.9)
p-value from ***χ***^**2**^ test of association		<0.01	0.7	<0.001
Sex				
• Male	63	26.3 (19.7–32.9)	28.6 (10.0–47.1)	30.0 (23.1–36.8)
• Female	108	20.4 (15.7–25.0)	31.3 (22.5–40.0)	27.4 (23.5–31.3)
p-value from ***χ***^**2**^ test of association		0.2	0.1	0.4
Location				
Rural	121	22.1 (18.1–26.1)	32.5 (22.3–42.8)	27.9 (24.1–31.6)
Urban	50	19.6 (15.5–23.6)	27.0 (13.0 = 40.9)	26.6 (22.9–30.3)
p-value from ***χ***^**2**^ test of association		0.7	<0.05	0.5
Functional domain*				
• Seeing	34	19.2 (15.1–23.3)	45.9 (14.8–70.9)	27.0 (23.2–30.8)
• Hearing	33	20.4 (16.4–24.4)	25.0 (7.2–42.8)	26.8 (23.1–30.5)
• Walking	38	22.3 (17.9–26.7)	17.9 (6.5–29.2)	29.0 (24.9–33.1)
• Self-care	36	22.5 (18.0–27.0)	37.1 (10.2–64.0)	29.0 (24.9–33.2)
• Communication	16	20.4 (16.0–24.8)	-	26.0 (22.0–30.0)
• Upper body	31	20.9 (16.6–25.3)	31.4 (2.3–60.6)	29.2 (25.0–33.3)
• Cognition	22	19.8 (15.5–24.1)	40.5 (14.7–66.3)	26.6 (22.7–30.5)
• Anxiety/depression	52	20.2 (16.0–24.3)	28.6 (19.9–37.2)	26.5 (22.8–30.3)

^*^Not mutually exclusive and so p-value could not be calculated

## Discussion

The SDGs aim to “Leave no one behind” and so have focussed international attention on being more inclusive. This directive provides strong rationale to ensure that access to WASH is inclusive of people with disabilities, in addition to the importance of respecting the fundamental rights of people with disabilities. [15] Although there is growing body of qualitative research suggesting that people with disabilities experience difficulties in accessing and using WASH, [5] this is not always reflected in the quantitative literature, [16–19] in part because tools available to assess WASH access are not sufficiently nuanced or detailed. This study was part of an effort to develop a tool for such an assessment, and test it within the context of a national survey in Guatemala.

Overall, our study showed few differences in WASH access between households that included a member with disabilities compared to those that did not, excepting the surprising finding that people with disabilities were more likely to live in households with an improved sanitation facility, which requires further exploration. These results are generally consistent with the existing literature that has not shown large differences in access to WASH between households that include people with disabilities and those that do not,[16–20] or else did not report on these links, implying that there were no associations.[21–24] In contrast, the current study showed differences in provision of sanitation and hygiene between people with and without disabilities when these were examined at the individual level using a more detailed survey tool. Fewer differences were apparent for water access in this context where the vast majority of people interviewed had water piped into their households. A previous study analysing data across five settings (Bangladesh– 2 settings, Malawi, Cameroon and India) also showed individual level differences in WASH access between people with and without disabilities, but no differences at the household level, supporting the pattern found in the current study.[19]

The lack of difference between people with and without disabilities in access to WASH at the household level is perhaps surprising, since both disability and lack of WASH are linked to poverty. [3, 10] Possible explanations are that disability often arises in older-age, and so may be less strongly related to household conditions, or that Guatemala has achieved relatively good WASH provision and consequently there is a ceiling effect. The individual level difficulties of people with disabilities in WASH-related activities detected shows that although WASH facilities may be available at the household level, they are not always accessible or adequate for people with disabilities, and this is also reflected in the qualitative literature.[5] These findings reinforce the argument for more nuanced individual level quantitative tools to assess WASH access and quality, such as the one used in this paper.

People with disabilities are not a homogenous group, and experience of WASH may vary among different population sub-groups. The current study shows that older people with disabilities were particularly likely to report difficulties in accessing WASH and that some aspects of WASH were worse for people with disabilities living in rural areas. No clear differences in WASH access were shown between men and women, or people with different impairment types, although the latter sub-group comparisons included relatively small numbers. Few other studies have investigated cross-cutting issues that influenced the relationship between WASH and disability. In the Malawi study, people with physical impairments reported more difficulties in physical access, while those with vision and hearing impairing reported more exclusion from WASH information.[6] Furthermore, women with disabilities had different WASH needs to men, not observed in the current study.

There are several strengths and limitations of this study that need to be considered when considering the findings. The study was large, conducted across Guatemala, and included in-depth WASH questions. The findings on high coverage of water pumped into households and widespread use of improved sanitation facilities were in keeping with recent data from Guatemala. [3, 25] In terms of limitations, no formal psychometric testing of the questionnaire was conducted, such as assessment of repeatability of results or comprehension of the questions or validation by objective measures of WASH. As an example, high numbers of both cases and controls reported coming into contact with urine when using the toilet, which was potentially surprising and may require exploration of the comprehension or wording of the question. However, a study in Nepal conducted qualitative interviews alongside the quantitative tool which will be able to explore some of these issues (results pending). The in-depth WASH tool was used on a sub-set of the total case-control study and so the numbers were small for some comparisons. Within Guatemala, most subjects had water piped to their compound so that the numbers of people reporting particular difficulties with accessing water was small, reducing the power to detect differences between cases and controls. The water module may, however, be relevant in other settings. Responses were given by the caregiver, rather than the person with disabilities, for children or for people with certain impairment types who were not able to answer for themselves (e.g. severe cognitive impairment). The present analyses show the quantitative differences in WASH access by disability status, but do not qualitatively explore the reasons for any differences including cultural aspects specific to Guatemala.

The tool presented in this study could help to improve the evidence base by capturing more detailed information about the WASH concerns of people with disabilities. It aimed to measure a lot of variability and diversity, yet with a limited number of questions. Consequently, several domains of difficulties in WASH were not included, notably questions around incontinence and WASH access outside of the household, which may need to be incorporated in future tools. The tool also relied on binary yes/no responses, and a scaled response may have been more discriminatory. The tool focussed on the appropriateness of existing WASH facilities, but did not explore what changes may be needed to make them more acceptable to people with disabilities (e.g. availability of hand rails in toilets) which would be helpful for planning purposes. Further work is needed on the current tool to ensure that it is comprehensive and has international applicability, and so it will need to be tested in more settings and revised. We welcome other researchers to use and improve the tool, and report on the results.

The inclusive WASH tool can help to identify key gaps where people with disabilities face exclusion and where solutions are needed. Policies around WASH are increasingly inclusive of people with disabilities, but this now needs to be translated into practice. [7] The evidence base on what is most effective to promote inclusion is currently lacking and needs to be improved, but a variety of strategies could be useful. Training about the WASH needs of people with disabilities is important to raise awareness and increase knowledge and this could target both WASH practitioners (e.g. Community Led Total Sanitation workers) and disability-related practitioners (e.g. Community Based Rehabilitation workers). [9] Within Guatemala, training may focus in particular on the needs of older people with disabilities, as they appear to experience greater WASH difficulties. Technical interventions could be scaled up, including adaptive technologies for accessible water and sanitation. Within Guatemala, the focus on inclusion in hygiene and sanitation appears more urgent than for water, where fewer concerns were identified. For all these interventions, it will be more cost-effective to plan for these adaptations from the start, rather than remodel existing facilities.

In conclusion, this research highlights the need for infrastructure and assistive devices that reduce pain, mitigate the risk of faecal contact, and provide appropriate bathing facilities for people with disabilities. Policies around WASH are increasingly inclusive of people with disabilities, but these now need to be translated into practice.[7] More detailed data are needed to generate information to highlight which WASH interventions are needed for people with disabilities, and allow assessment of their effectiveness, and the tool used in this paper may provide a useful starting point for generating this information.

## Supporting information

S1 Table Items contributing towards the water, sanitation and hygiene scores. (DOCX)

S2 Table Age and sex distribution of the national population and study sample. (DOCX)
