# Supplementary material for: Exploring the links between water, sanitation and hygiene and disability; Results from a case-control study in Guatemala
Source: PLoS One. 2018 Jun 1;13(6):e0197360. doi: 10.1371/journal.pone.0197360 (PMC5983415; doi:10.1371/journal.pone.0197360)
Supplement: S1 Table — (DOCX) [file pone.0197360.s001.docx]

**Supporting TABLE 1: Items contributing towards the Water, Sanitation and Hygiene Scores**

| WASH domain | Items included (Yes/no response options) |
| --- | --- |
| Water | - Cannot collect water for drinking - Is not able to access water when needed - Needs assistance to access water - Cannot use the same water source as other household members - Cannot collect the same quantity as household members - Causes additional pain to collect water - Is afraid of physical or verbal violence when collecting water. |
| Sanitation | - Uses a different toilet facility to other members of the household - Causes additional pain to use facility - Has less privacy for defecation than other members of the household - Is not usually able to use the toilet without clothes or self coming into contact with faeces - Is not usually able to use the toilet without clothes or self coming into contact with urine - Is not usually able to use the toilet without assistance from another person - Makes changes to daily routine or practices in use of the toilet. |
| Hygiene | - Needs help for bathing - Uses a different place for bathing to other household members - Causes additional pain to bathe - Has less privacy for bathing than other members of the household - Is afraid of physical or verbal violence when bathing - Comes into contact with dirt or dirty water whilst bathing - Is not able to wash hands without help from others - Is not able to locate and use soap and other cleansing materials without help from others. |
