# Supplementary material for: Exploring the links between water, sanitation and hygiene and disability; Results from a case-control study in Guatemala
Source: PLoS One. 2018 Jun 1;13(6):e0197360. doi: 10.1371/journal.pone.0197360 (PMC5983415; doi:10.1371/journal.pone.0197360)
Supplement: S2 Table — (DOCX) [file pone.0197360.s002.docx]

**Supporting TABLE 2: Age and sex distribution of the national population and study sample^*^**

|  | **Male** | | **Female** | | **Total** | |
| --- | --- | --- | --- | --- | --- | --- |
| **Age group (years)** | **National** | **Sample** | **National** | **Sample** | **National** | **Sample**** |
| **0-14** | 2711683 (36%) | 2216 (37%) | 2608295 (34%) | 2220 (31%) | 5,319,978 (35%) | 4,146 (34%) |
| **15-24** | 1663484 (22%) | 1323 (22%) | 1647749 (21%) | 1582 (22%) | 3,311,233 (22%) | 2,905 (22%) |
| **25-54** | 2425931 (32%) | 1772 (29%) | 2666790 (35%) | 2435 (35%) | 5,092,721 (34%) | 4,208 (32%) |
| **55-64** | 377,672 (5%) | 325 (5%) | 416,939 (5%) | 413 (6%) | 794,611 (5%) | 738 (6%) |
| **65+** | 311,165 (4%) | 397 (6%) | 360,280 (5%) | 409 (6%) | 671,445 (4%) | 806 (6%) |
| **Total** | 7489935 (49%) | 6033 (46%) | 7700053 (51%) | 7,039 (54%) | 15189988 | 13,073 |

*This table is reproduced from the ENDIS report. [11]

**Data on sex missing for 1 person; Source of national estimates: CIA world Fact book, 2016
